# Wasting, underweight and stunting among children with congenital heart disease presenting at Mulago hospital, Uganda

**DOI:** 10.1186/s12887-017-0779-y

**Published:** 2017-01-11

**Authors:** Anthony Batte, Peter Lwabi, Sulaiman Lubega, Sarah Kiguli, Kennedy Otwombe, Lucy Chimoyi, Violette Nabatte, Charles Karamagi

**Affiliations:** 1 Child Health and Development Centre, Makerere University College of Health Sciences, P.O.Box 6717, Kampala, Uganda; 2 Uganda Heart Institute, P.O. Box 7051, Kampala, Uganda; 3 Department of Pediatrics and Child Health, Makerere University College of Health Sciences, P.O.Box 7072, Kampala, Uganda; 4 Perinatal HIV Research Unit, Faculty of Health Sciences, University of the Witwatersrand, Johannesburg, South Africa; 5 The Aurum Institute, Johannesburg, South Africa; 6 Nakaseke Hospital, Nakeseke, Uganda; 7 Clinical Epidemiology Unit, Makerere University College of Health Sciences, P.O.Box 7072, Kampala, Uganda

**Keywords:** Congenital heart disease, Malnutrition, Uganda, Africa

## Abstract

**Background:**

Children with congenital heart disease are at increased risk of malnutrition. The aim of this study was to describe the prevalence of wasting, underweight and stunting among children with congenital heart disease attending Mulago National Referral Hospital, Uganda.

**Methods:**

A cross-sectional study among 194 children aged 0–15 years was conducted between August 2013 and March 2014. Anthropometric measurements and clinical assessments were carried out on all children. Anthropometric z-scores based on WHO 2007 reference ranges were generated for each child. Weight-for-height z-scores were generated for children 0–5 years, weight-for-age z-scores for children 0–10 years, and height-for-age and BMI-for-age z-scores for all children. Risk factors associated with malnutrition were determined by Poisson regression.

**Results:**

One hundred and forty five (74.7%) children were aged 0–5 years; and 111 of 194 (57.2%) were female. Forty five of 145 (31.5%) children aged 0–5 years were wasted; 77 of 181 (42.5%) children aged 0–10 years were underweight; 88 of 194 (45.4%) children were stunted; and 53 of 194 (27.3%) children were thin (BMI for age z score < −2). Moderate to severe anaemia (RR 1.11, 95% CI: 1.01–1.22) and moderate to severe heart failure (RR 1.24, 95% CI: 1.13–1.36) were associated with wasting and underweight respectively. Stunting was associated with moderate to severe heart failure (RR 1.11, 95% CI: 1.01–1.21) while thinness was associated with moderate to severe heart failure (RR 1.12, 95% CI: 1.04–1.21) and moderate to severe anaemia (RR 1.15, 95% CI: 1.06–1.25).

**Conclusion:**

Malnutrition is common in children with congenital heart disease, and is associated with anaemia and heart failure. There is need to integrate strategies to identify and manage malnutrition during the care of children with congenital heart disease.

## Background

Children with congenital heart disease (CHD) are at an increased risk for wasting, underweight and stunting [1–3]. Malnutrition among these children increases their risk to infections and the risk of death even after corrective surgery [4]. In addition, malnutrition leads to poor growth in children which is associated with delayed mental development, poor school performance and reduced intellectual capacity [4, 5]. These outcomes significantly impair economic productivity in adult life.

Risk factors for malnutrition among children with CHD include heart failure, cyanosis, multiple heart defects, delayed corrective surgery, anaemia and pulmonary hypertension [1, 3, 6]. Furthermore, in children with CHD, poor nutrition is also attributed to inadequate nutritional intake due to feeding difficulties and the increased energy expenditure among these children [7]. In developing countries, corrective surgery for congenital heart defects is delayed and this increases the likelihood of the children developing malnutrition [8].

In Uganda, childhood malnutrition is a public health problem with 33% of children aged under 5 years being stunted, 14% underweight and 5% wasted [9]. Thus malnutrition in children with CHD in Uganda is likely to be common and of multi-factorial origin. A clear understanding of the complex nature of malnutrition in children with CHD is essential to design strategies that will improve outcomes in these children. This study was therefore conducted to determine the prevalence of malnutrition in children with congenital heart disease and the factors associated with wasting, underweight and stunting.

## Methods

### Study setting and population

The study was carried out at Mulago National Referral Hospital and Uganda Heart Institute, Kampala, from August 2013 to March 2014. Participants were 194 children aged 0–15years with congenital heart disease diagnosed by echocardiography. They were part of a previous study which assessed arrhythmias among children; and the details of the methods and the different heart diseases for each child are described elsewhere [10].

### Variables

Data were collected on socio-demographic characteristics and clinical assessments of the children. Weight was measured using a SECA® weighing scale and readings were recorded to the nearest 0.1 kg. Height was measured using a stadiometer (measuring board) and readings recorded to the nearest 0.5 cm. Heights for children below 2 years was taken when the children were lying down while older children had their heights taken when standing. We used the World Health Organisation (WHO) z-scores to classify the nutritional status of the children [11]. A child was classified as underweight if the weight-for-age WHO z-score was < −2 SD, wasted if weight-for-height WHO z-score was < −2 SD (for children aged 0–5 years), thin if BMI-for-age WHO z-score was < −2 SD and stunted if height-for-age z-score was < −2 SD.

All children were assessed for heart failure and the severity of heart failure was graded using the modified Ross score; children were grouped into two categories based on a Ross score 0–6 (mild or no heart failure) and a Ross score >6 (moderate to severe heart failure) [12, 13].

Hemoglobin levels of the children were recorded from complete blood counts that were performed using Celltac E MEK-7222 automatic hematology analyzer. Grading of the severity of anaemia (mild, moderate and severe) was done according to WHO age specific reference ranges [14]. An HIV test was performed on each child using Alere™ HIV-1/2 Determine® as a screen, followed by STAT-PAK® HIV-1/2; the Uni-Gold™ Recombigen® for confirmation. HIV infection in children aged under 18 months who were HIV positive by serology test was confirmed using the HIV DNA PCR test.

### Data analysis

Age was categorized into three groups: 0–5, 6–10 and 11–15 years. The demographic and clinical characteristics were stratified by age. Continuous anthropometric measures were stratified using a z-score of < −2 as a cut-off for malnutrition. Overall frequencies were determined for all categorical measures followed by stratification by age. Medians and interquartile ranges of the anthropometric measures were determined overall and by age.

Poisson regression with robust error variances was used to determine factors associated with malnutrition both at the univariate and multivariate level. All the variables with at least one factor having a *p*-value < 0.2 at the univariate level were considered for entry into the multivariate model controlling for age. Statistical analysis was performed using SAS/STAT software version 9.4 (SAS Institute Inc., Cary, NC, USA).

## Results

### Demographic characteristics of participants

One hundred and forty five of 194 children (74.7%) were aged 0–5 years and 111 of 194 (57.2%) were female. One hundred and thirty eight of 194 children (71.1%) had acyanotic heart disease but only 4 (2.1%) had surgically corrected heart defects (Table 1). Two children (1%) were HIV positive.

**Table 1:** Demographic and clinical characteristics

Variable	Overall	0-5 years (*n* = 145)	6-10 years (*n* = 36)	11-15 years (*n* = 13)
Gender				
Male (%)	83 (42.8)	65 (44.8)	14 (38.9)	4 (30.8)
Female (%)	111 (57.2)	80 (55.2)	22 (61.1)	9 (69.2)
Classification of heart disease				
Cyanotic (%)	56 (28.9)	39 (26.9)	13 (36.1)	4 (30.8)
Acyanotic (%)	138 (71.1)	106 (73.1)	23 (63.9)	9 (69.2)
Heart disease surgically corrected				
Yes (%)	4 (2.1)	2 (1.4)	1 (2.8)	1 (7.7)
No (%)	190 (97.9)	143 (98.6)	35 (97.2)	12 (92.3)
Heart failure				
Moderate/ Severe Heart failure (%)	78 (40.2)	58 (40.0)	16 (44.4)	4 (30.8)
Mild/ no Heart failure (%)	116 (59.8)	87 (60.0)	20 (55.6)	9 (69.2)
Anaemia				
Moderate/ Severe anaemia (%)	68 (35.6)	58 (40.8)	8 (22.2)	2 (15.4)
Mild/ No Anaemia (%)	123 (64.4)	84 (59.2)	28 (77.8)	11 (84.6)

### Proportion of children wasted, underweight and stunted

Overall, 45 of 145 (31.5%) children aged 0–5 years had weight-for-height z-score < −2 (wasted). The median weight-for-height z-score among these children was −0.89 (IQR: −2.46,-0.10). Of the children aged 0–10 years, 77 of 181 (42.5%) had weight-for-age z-scores < −2 (underweight) and the median weight-for-age z-score among these children was −1.69 (IQR-3.07,-0.69). Fifty three of 194 children (27.3%) had a BMI-for-age z-score < −2 (thin) with a median BMI-for-age z-score of −0.72 (IQR −2.21, 0.17). The number of stunted children was 88 out of 194 (45.4%) with a median height-for-age z-score of −1.67 (IQR −3.21,-0.51). These findings are summarised in Table 2.

**Table 2:** Proportion of children wasted, underweight and stunted

Variable	Overall	0–5 years (*n* = 145)	6–10 years (*n* = 36)	11–15 years (*n* = 13)
Weight-for-Height z-score				
< −2 (%)	45 (31.5)	45 (31.5)	N/A	N/A
≥ 2 (%)	98 (68.5)	98 (68.5)	N/A	N/A
Median WHZ (IQR)	*n* = 143; −0.89 (−2.46,0.10)	*n* = 143; −0.89 (−2.46,0.10)	N/A	N/A
Weight-for-age z-score				
< −2 (%)	77 (42.5)	65 (44.8)	12 (33.3)	N/A
≥ 2 (%)	104 (57.5)	80 (55.2)	24 (66.7)	N/A
Median WAZ (IQR)	*n* = 181; −1.69 (−3.07,-0.69)	*n* = 145; −1.83 (−3.36,-0.62)	*n* = 36; −1.54 (−2.17,-0.77)	N/A
Height-for-age z-score				
< −2 (%)	88 (45.4)	70 (48.3)	14 (38.9)	4 (30.8)
≥ 2 (%)	106 (54.6)	75 (51.7)	22 (61.1)	9 (69.2)
Median HAZ (IQR)	*n* = 194; −1.67 (−3.21,-0.51)	*n* = 145; −1.91 (−3.34,-0.58)	*n* = 36; −1.62 (−2.47,-0.52)	*n* = 13; −0.93 (−2.57,-0.47)
BMI for age z-score				
< −2 (%)	53 (27.3)	46 (31.7)	4 (11.1)	3 (23.1)
≥ 2 (%)	141 (72.7)	99 (68.3)	32 (88.9)	10 (76.9)
Median BMIZ (IQR)	*n* = 194; −0.72 (−2.21,0.17)	*n* = 145; −0.74 (−2.41,0.33)	*n* = 36; −0.61 (−1.53,-0.15)	*n* = 13; −0.81 (−1.89,0.29)

*N/A* z-scores for children in this age group cannot be generated using the WHO 2007 z scores

### Factors associated with malnutrition

Among children under 5 years, children with moderate/severe anaemia (RR: 1.11, 95% CI: 1.01–1.22) had a higher risk of being wasted after controlling for age and heart failure (Table 3). Among children aged 0–10 years, moderate/severe heart failure was associated with being underweight after adjusting for age and anaemia (RR: 1.24, 95% CI: 1.13–1.36) (Table 4). After controlling for age and anaemia, children with moderate/severe heart failure had a higher risk of stunting (RR 1.11, 95% CI 1.01–1.21) (Table 5). After controlling for age, children with moderate/severe heart failure (RR: 1.12, 95% CI: 1.04–1.21) and moderate/severe anaemia (RR: 1.15, 95% CI: 1.06–1.25) had a higher risk of thinness (Table 6).

**Table 3:** Factors associated with wasting among children under 5 years (Weight-for-Height z-score < −2)

Variables	Univariate		Multivariate	
	RR (95% CI)	*P*-Value	RR (95% CI)	*P*-Value
Gender				
Male	Ref			
Female	0.99 (0.90–1.09)	0.844		
Age (years)	0.96 (0.93–0.99)	0.009	0.97 (0.94–1.002)	0.068
Cyanotic heart disease				
No	Ref			
Yes	0.97 (0.88–1.07)	0.598		
Heart failure				
No/mild heart failure	Ref		Ref	
Moderate/ severe heart failure	1.13 (1.03–1.25)	0.012	1.1 (0.997–1.22)	0.058
Anaemia				
No/ mild anaemia	Ref		Ref	
Moderate/severe anaemia	1.13 (1.02–1.24)	0.017	1.11 (1.01–1.22)	0.03

**Table 4:** Factors associated with underweight

Variables	Univariate		Multivariate	
	RR (95% CI)	*P*-Value	RR (95% CI)	*P*-Value
Gender				
Male	Ref			
Female	0.99 (0.91–1.09)	0.905		
Age (years)				
0–5	Ref		Ref	
6–10	0.93 (0.84–1.04)	0.187	0.93 (0.84–1.03)	0.206
11–15	N/A			
Cyanotic heart disease				
No	Ref			
Yes	1.03 (0.93–1.15)	0.537		
Heart failure				
No/mild heart failure	Ref		Ref	
Moderate/ severe heart failure	1.24 (1.13–1.36)	<0.001	1.24 (1.13–1.36)	<0.001
Anaemia				
No/ mild anaemia	Ref		Ref	
Moderate/severe anaemia	1.09 (0.99–1.20)	0.092	1.09 (0.99–1.20)	0.086

**Table 5:** Factors associated with stunting

Variables	Univariate		Multivariate	
	RR (95% CI)	*P*-Value	RR (95% CI)	*P*-Value
Gender				
Male	Ref			
Female	0.995 (0.91–1.09)	0.919		
Age (years)				
0–5	Ref		Ref	
6–10	0.94 (0.84–1.05)	0.295	0.94 (0.84–1.05)	0.292
11–15	0.90 (0.77–1.05)	0.175	0.91 (0.78–1.07)	0.264
Cyanotic heart disease				
No	Ref			
Yes	1.01 (0.91–1.12)	0.85		
Heart failure				
No/mild heart failure	Ref		Ref	
Moderate/ severe heart failure	1.11 (1.01–1.22)	0.026	1.11 (1.01–1.21)	0.037
Anaemia				
No/ mild anaemia	Ref		Ref	
Moderate/severe anaemia	1.07 (0.97–1.18)	0.161	1.06 (0.96–1.17)	0.231

**Table 6:** Factors associated with low BMI z-score

Variables	Univariate		Multivariate	
	OR (95% CI)	*P*-Value	OR (95% CI)	*P*-Value
Gender				
Male	Ref			
Female	1.01 (0.94–1.08)	0.825		
Age (years)				
0–5	Ref		Ref	
6–10	0.89 (0.83–0.96)	0.0013	0.91 (0.84–0.97)	0.006
11–15	0.95 (0.83–1.09)	0.47	0.993 (0.86–1.14)	0.91
Cyanotic heart disease				
No	Ref			
Yes	0.97 (0.90–1.04)	0.40		
Heart failure				
No/mild heart failure	Ref		Ref	
Moderate/ severe heart failure	1.12 (1.03–1.21)	0.006	1.12 (1.04–1.21)	0.004
Anaemia				
No/ mild anaemia	Ref		Ref	
Moderate/severe anaemia	1.16 (1.07–1.27)	0.0005	1.15 (1.06–1.25)	0.001

## Discussion

Our study showed that malnutrition in children with congenital heart disease is high. The prevalence of wasting, underweight, thinness and stunting was 31.5%, 42.5%, 27.3% and 45.4% respectively. Malnutrition was more frequent among children with moderate to severe heart failure and moderate to severe anaemia.

The prevalence of wasting among children aged under 5 years with congenital heart disease in our study was higher than the 7.5% global prevalence of wasting in children under 5 years [15]. In addition, our findings show that children with congenital heart disease are six times more likely to be wasted compared to their counterparts in Uganda [9]. Our study found a high proportion of underweight, stunted and thin children relative to those previously described globally [15, 16] and in Uganda [9, 17]. Other studies have also demonstrated that children with congenital heart disease are at higher risk of malnutrition compared to those without heart disease [1, 18, 19].

Okoromah and colleagues reported prevalence of wasting, stunting and underweight of 41.1%, 28.8% and 20.5% respectively in children with congenital heart disease attending an outpatient tertiary hospital in Nigeria [1]. Although they noted a higher prevalence of wasting, and the proportions of stunted and underweight children were lower than those seen in our study. Furthermore, in comparison to our study, a higher prevalence of malnutrition among children with congenital heart disease was described in India; wasting (55.9%) and underweight (59%) [20]. Lower prevalence of wasting (23.8%) and underweight (14.3%) was reported in Egypt among children under 6 years of age [21]. These studies indicate that the prevalence of malnutrition is high in children with congenital heart disease in various countries. However, heterogeneity exists from country to country implying differences in risk factors for malnutrition among children, in the different settings.

Similar to our findings, various studies have reported an association between malnutrition and heart failure in children with congenital heart disease [1, 20, 21] which is attributed to increased metabolic needs of these children yet they have low energy intake due to their inability to tolerate a high volume of feeds [7, 22].

Anaemia has also been reported to be associated with malnutrition in children with congenital heart disease [1, 21]. This is similar to our findings where children with moderate or severe anaemia were more likely to be wasted and thin. Children with malnutrition commonly have anaemia which is attributed to bone marrow hypoplasia, iron, vitamin B12, vitamin A and folate deficiency [23, 24]. Even though we did not assess for these causes of anaemia in our study, we think that these could explain the association between anaemia and malnutrition among children with congenital heart disease noted from our study.

Delayed time to surgical correction of the heart defects has been associated with malnutrition among the children with congenital heart disease [1, 3, 20]. In our study, only 4 children had received surgical correction of their heart defects, and therefore we could not assess this relationship due to small numbers. There was no significant association between cyanotic heart disease and malnutrition, a finding that is contrary to other studies. It may be that a different outcome would have been achieved by a larger sample size.

Although, Uganda has a number of policies and guidelines on promotion of nutrition among children, there is limited emphasis on congenital heart diseases as an underlying risk factor for malnutrition [25, 26]. Hence there is a need to develop interventions such as early surgical repair for heart defects and institutionalisation of other measures to prevent and manage malnutrition in these children.

### Limitations

We did not assess other factors that are known to contribute to malnutrition in these children with congenital heart disease such as genetic disorders [27], pulmonary hypertension [3] and social economic status of the households. However, socio-economic status is better assessed in community studies than hospital based studies.

Children with cyanotic congenital heart disease tend to have polycythemia resulting in higher haemoglobin levels [28]. In our study, we used the WHO age specific reference haemoglobin levels to define anaemia [14]. In children with cyanotic heart disease, these reference ranges may not be the best markers for anaemia. However, there are no standardised haemoglobin reference levels for these children and the variations in their haemoglobin levels are not uniform. The sample size of the children in our study is also relatively small but this may not have significantly affected our results.

## Conclusion

Children with congenital heart disease had high prevalence rates of wasting, underweight, stunting and thinness. Wasting was associated with moderate to severe anaemia. Stunting and underweight were associated with moderate to severe heart failure, while thinness was associated with moderate to severe heart failure and moderate to severe anaemia. With these high rates of malnutrition in children with congenital heart disease, there is need to integrate strategies to identify and manage malnutrition during their care.

## Abbreviations

BMIZ: Body mass index-for-age z score
CHD: Congenital heart disease
HAZ: Height-for-age z score
WAZ: Weight-for-age z score
WHO: World Health Organisation
WHZ: Weight-for-height z score
